# Inhalable Microparticles Embedding Calcium Phosphate Nanoparticles for Heart Targeting: The Formulation Experimental Design

**DOI:** 10.3390/pharmaceutics13111825

**Published:** 2021-11-01

**Authors:** Eride Quarta, Fabio Sonvico, Ruggero Bettini, Claudio De Luca, Alessandro Dotti, Daniele Catalucci, Michele Iafisco, Lorenzo Degli Esposti, Gaia Colombo, Giovanna Trevisi, Dimitrios M. Rekkas, Alessandra Rossi, Tin Wui Wong, Francesca Buttini, Paolo Colombo

**Affiliations:** 1 Food and Drug Department, University of Parma, Parco Area delle Scienze 27/A, 43124 Parma, Italy; eride.quarta@studenti.unipr.it (E.Q.); fabio.sonvico@unipr.it (F.S.); ruggero.bettini@unipr.it (R.B.); alessandra.rossi@unipr.it (A.R.); 2 PlumeStars Srl., c/o Food & Drug Department, Parco Area delle Scienze 27A, 43124 Parma, Italy; 3 Fin-Ceramica Faenza SPA, Via Granarolo 177/3, 48018 Faenza, Italy; cdeluca@finceramica.it (C.D.L.); alessandro.dotti@finceramica.it (A.D.); 4 IRCCS Humanitas Research Hospital, 20089 Rozzano, Italy; daniele.catalucci@cnr.it; 5 Institute of Genetic and Biomedical Research (IRGB)-UOS Milan, National Research Council (CNR), 20138 Milan, Italy; 6 Institute of Science and Technology for Ceramics (ISTEC), National Research Council (CNR), Via Granarolo 64, 48018 Faenza, Italy; michele.iafisco@istec.cnr.it (M.I.); lorenzo.degliesposti@istec.cnr.it (L.D.E.); 7 Department of Life Sciences and Biotechnology, University of Ferrara, Via Fossato di Mortara 17/19, 44121 Ferrara, Italy; clmgai@unife.it; 8 Institute of Materials for Electronics and Magnetism (IMEM), National Research Council (CNR), Parco Area delle Scienze 37/A, 43124 Parma, Italy; giovanna.trevisi@imem.cnr.it; 9 Department of Pharmacy, National and Kapodistrian University of Athens, 15784 Zografou, Athens, Greece; rekkas@pharm.uoa.gr; 10 Non-Destructive Biomedical and Pharmaceutical Research Centre, Smart Manufacturing Research Institute, Universiti Teknologi MARA, Puncak Alam 42300, Selangor, Malaysia; wongtinwui@uitm.edu.my

**Keywords:** calcium phosphate nanoparticles, microparticles embedding nanoparticles, design of experiment, pulmonary delivery

## Abstract

Inhalation of Calcium Phosphate nanoparticles (CaPs) has recently unmasked the potential of this nanomedicine for a respiratory lung-to-heart drug delivery targeting the myocardial cells. In this work, we investigated the development of a novel highly respirable dry powder embedding crystalline CaPs. Mannitol was selected as water soluble matrix excipient for constructing respirable dry microparticles by spray drying technique. A Quality by Design approach was applied for understanding the effect of the feed composition and spraying feed rate on typical quality attributes of inhalation powders. The in vitro aerodynamic behaviour of powders was evaluated using a medium resistance device. The inner structure and morphology of generated microparticles were also studied. The 1:4 ratio of CaPs/mannitol led to the generation of hollow microparticles, with the best aerodynamic performance. After microparticle dissolution, the released nanoparticles kept their original size.

## 1. Introduction

Rusconi et al. 2016 [1] have demonstrated that the cell-penetrating mimetic peptide (R7W-MP) targeting the Ca_v_β2 cytosolic subunit of the L-type calcium channel (LTCC) restored cardiac contractility in the pathological conditions of LTCC-based cardiomyopathy. However, the active Mimetic Peptide (MP) without the R7W cell-penetrating sequence loses the capability to enter the cardiac cells.

In a follow-up paper, the in vivo administration by nebulization of MP-loaded CaPs enabled their internalization into cardiomyocytes and restored cardiac function in a mouse model of cardiomyopathy [2]. Thus, this heart targeted treatment took advantage of the pulmonary administration route for cardiac accumulation of drug loaded CaPs.

However, drug loaded CaPs (hydrodynamic mean diameter < 200 nm) present complexity for the administration by inhalation due to difficulty to control their nano size before and after delivery. One technological solution to achieve lung deposition of nanoparticles is their transformation into a microparticulate dry powder to inhale from a device [3]. In this case, their physical stability is improved, the dose control is more predictable, and the product could be used in home or care settings.

The research hypothesis underlying this work was to take advantage of the transient size of respirable microparticles obtained by embedding the nanoparticles in water-dissolving structures. Therefore, the aim was to study how to construct by spray drying inhalable microparticles embedding CaP nanoparticles for a deep lung deposition. Mannitol was selected as a soluble carrier, creating a matrix in which CaPs are homogeneously dispersed. Thus, microparticles in the respirable size range were prepared by spray-drying nanoparticle dispersions in an aqueous mannitol solution.

A Design of Experiments (DoE) toolbox was adopted for the identification of the Critical Process Parameters (CPPs) affecting the Critical Quality Attributes (CQAs) of respirable dry powders made of CaP nanoparticles embedded in microparticles [4]. Drying process yield, powder moisture content, microparticle size distribution, microparticle aerodynamic performance and size of released CaP nanoparticles were the CQAs [5].

A basic full factorial design was chosen to study the effects of selected input variables, i.e., the feed rate and CaPs and mannitol concentrations in the dispersion to spray dry.

## 2. Materials and Methods

### 2.1. Materials

CaP nanoparticles in water dispersion were prepared by a continuous flow process as reported in the paper of Degli Esposti et al. [6]. A pilot scale batch of three litres of unloaded CaPs was manufactured. The nanoparticle in dispersion had a concentration of 7.0 mg/mL. CaP nanoparticles had a Z-average diameter of 80 ± 15 nm, a polydispersity index (PdI) of 0.2, and a negative ζ-potential of −25 ± 2 mV. The Ca/P molar ratio, determined by inductively coupled plasma-optical emission spectrometry analysis, was 1.6 ± 0.2, in agreement with the typical value of nanocrystalline hydroxyapatite [7].

Mannitol (Ph.Eur.) was used as an excipient to manufacture microparticles.

### 2.2. Methods

#### 2.2.1. DoE for the Manufacturing of Microparticles Embedding Nanoparticles

The manufacturing of spray-dried microparticles embedding unloaded CaPs was studied by applying a two-level full factorial design with 3 factors (2^3^), investigating all the possible combinations between the selected levels. This factorial design was kept as simple as possible for an initial screening of the factors effect on the quality of the final product. Therefore, a process manufacturing parameter and two critical material attributes were selected. These parameters relate to the future industrial production of powders and the structure of the microparticles. All statistically significant main effects and interactions of the input factors on the CQAs of the dry powder for inhalation were identified. In addition, three centre points were added to check for a possible curvature. The design was of resolution V, meaning that there is no confounding effect between the main factors and their interactions. Thus, a total 11 experiments was generated and executed in a randomized way. The evaluation of the responses was performed both with relevant statistical graphs (Pareto charts) and ANOVA test (confidence interval: 95%, desired power: 80–90%, minimum variance: 0.0909). The significance of the mathematical model, as well as the impact of each input parameter on CQAs, were determined using the Design-Expert Software^®^ Version 12 (Stat-Ease Inc., Minneapolis, MN, USA).

Practically, spray-dried microparticles for inhalation embedding unloaded CaPs were manufactured using a Büchi Mini Spray Dryer B-290 (Büchi Laboratory Equipment, Flawil, Switzerland). The aqueous dispersions to be dried were prepared starting from diluted nanoparticle dispersions to obtain the CaPs concentration reported in Table 1. Mannitol was dissolved in the CaPs dispersion to achieve the concentrations reported in Table 1. The dispersions were kept under magnetic stirring throughout the drying process. The spray drying feed rate was set accordingly to the values in Table 1. The other spray drying operating parameters were fixed as follows: inlet temperature 125 °C, air flow rate 600 L/h, aspiration 35 m^3^/h and nozzle 0.7 mm.

The yield of the process was calculated using Equation (1):

(1) $$Yield=\frac{W_{A}}{W_{T}}*100$$

where W_A_ is the weight of the powder recovered from the collection vessel and the cyclone, while W_T_ is the weight of total solids in the dispersion to dry. Finally, the dry powders recovered were stored in a sealed glass vial at 25 °C.

#### 2.2.2. Microparticle Morphology by Scanning Electron Microscopy (FESEM-FIB)

The microparticle morphology, surface texture and internal structure were investigated by a Field Emission Scanning Electron Microscope equipped with a Ga Focused Ion Beam (FIB) Auriga Compact (Zeiss, Jena, Germany). The samples were prepared by dispersing 1–2 mg of microparticles directly on the carbon tape placed on the aluminium stubs. The surface morphology and texture of the microparticles were investigated in plan-view by using a 1 kV electron beam acceleration voltage. Such a low value allowed to analyze the microparticles without the need for metallization. To study the internal structure of microparticles embedding CaPs, the samples were tilted at 54° with respect to plan-view zero-tilt condition. The particles were cut by FIB with an operating voltage of 30 kV and a low current of 50 pA. The images of the microparticles cross-sections were acquired by using the same operating conditions selected for plan-view analysis. All the images were acquired with the Everhart-Thornley detector for secondary electrons.

#### 2.2.3. Microparticle Size Distribution by Laser Diffraction

The particle size distribution (PSD) of powders was measured using the diffractometer Spraytec (Malvern Instruments Ltd., Worcestershire, UK) equipped with a 300 mm focal lens. Briefly, 10 mg of powder was dispersed in 10 mL of cyclohexane (VWR International, France) containing 0.1% *w/v* of Span 85 (Merck KGaA, Darmstad, Germany). The dispersion was placed in an ultrasonic bath for 1 min and particle size distribution was measured with 5% threshold obscuration.

#### 2.2.4. In Vitro Aerodynamic Assessment of Microparticles by Fast-Screening Impactor (FSI)

Next, 40 mg of powder was introduced into HPMC QUALI-V I size 3 capsules (Qualicaps, Madrid, Spain). The loaded capsule was then inserted into the holder chamber of the RS01^®^ Medium Resistance Monodose Dry Powder Inhaler, (Plastiape, Lecco, IT). An abbreviated impactor (Fast Screening Impactor (FSI), Copley Scientific, Nottingham, UK) was employed for the powder separation in two fractions. In consideration of the monolithic structure of the mannitol microparticles, incorporating drug-free CaP nanoparticles, the adoption of the filter weighing procedure to determine the FPF values of powder aerosol with FSI was considered sufficiently accurate. The mass of fine microparticles weighed was in the order of tens of milligrams. Thus, a glass fibre filter, pre-weighed, was placed on the FSI Fine Fraction Collector (FFC) stage to recover the particles with an aerodynamic diameter lower than 5 μm. The assembled FSI was connected to a vacuum pump (Erweka GmbH, Langen, DE). The dry powder inhaler with capsule was weighed and the capsule pierced. An aspiration flow rate of 60 L/min was applied for 4 s (Copley Scientific, Nottingham, UK) at a pressure drop of 4 kPa over the inhaler, to aerosolize the capsule content. After aerosolization, the FFC filter holder was disassembled and the filter carefully transferred into a plastic Petri dish. Then, the filter, as well as the system device-capsule, were weighed in a balance (limit 10 mcg). The difference between the filter weight before and after device actuation provides the Emitted Dose (ED) which expresses the amount of powder that left the device. The ratio between the ED and metered dose defines the Emitted Fraction (EF). Finally, the weight of the powder deposited on the FFC filter is the Fine Particle Dose (FPD) of the powder (aerodynamic diameter < 5 mm), while the ratio of the FPD with the ED gives the Fine Particle Fraction (FPF). ED, EF, FPD and FPF are indicators of the powder aerodynamic performance.

#### 2.2.5. Density Measurement

The true density of powders was measured with AccuPyc II 1340 gas pycnometer (Micromeritics Instrument Corporation, Norcross, GA, USA). Bulk and tapped densities were determined following Ph. Eur. 10th ed. Three measurements were performed for each sample.

#### 2.2.6. Thermogravimetric Analysis (TGA)

Thermogravimetric analysis for the calculation of the residual solvent content of powders was performed with a TGA equipment (METTLER Toledo, Worthington, OH, USA). For the purpose, 3–5 mg of each spray-dried powder was accurately weighed in a 70 μL alumina pan with a pierced cover (crucibles). The analysis was done by heating the sample under a flux of dried nitrogen (80 mL/min) from 25 °C to 150 °C at a rate of 10 °C/min.

#### 2.2.7. Dimensional Analysis and Surface Charge of Released Nanoparticles by Dynamics Light Scattering

A protocol for CaP nanoparticle release from microparticles was established to evaluate the influence of the spray drying procedure and microparticle composition on the properties of released CaPs. An amount of powder was dissolved with gentle shaking in ultrapure water. Z-average diameter, Polydispersity Index and ζ-potential were determined using Dynamic Light Scattering (DLS) Zetasizer Nano ZS (Malvern Instruments Ltd., Worcestershire, UK).

## 3. Results and Discussion

### 3.1. DoE Analysis of Microparticle Manufacturing

The objective of the DoE process was to study the production of microparticulate powders and to assess in vitro their features for the deposition in the deep lung and for the nanoparticle release in a size comparable to the original. Mannitol was selected as the carrier that could provide an efficient nanoparticles re-dispersion of nanoparticles after excipient dissolution.

CaPs and mannitol concentrations were the formulation factors selected. In fact, the aerodynamic behaviour of microparticles depends on the solid structure obtained following solvent evaporation. In consideration of its relevance for the industrial manufacturing time, the feed rate of the liquid to dry was selected as a process parameter.

To evaluate the prepared powders within the quality by design framework, the CQAs considered were the process yield, moisture content, microparticle size distribution (median volume diameter, D_v50_) and aerodynamic performance, expressed as Emitted Dose and Fine Particle Dose. The hydrodynamic diameter (d_H_) of the nanoparticles, released upon microparticle dissolution, was measured as well.

Thus, a full factorial experimental design was chosen by combining three factors at two levels. In total, eleven experiments (eight factorials and three central repetitions) were generated in a randomized way to avoid bias. The process and composition factors with their corresponding levels are reported in Table 2.

The corresponding CQAs of the produced microparticles, replicated three times, are also shown in Table 2 for all the eleven formulations prepared. The low, middle, and high levels of the factors are designated with −1, 0, and +1, respectively. The three centre points of experiments correspond to Runs 4, 8, and 10.

#### 3.1.1. Yield of the Spray Drying Process

**Figure 1:**
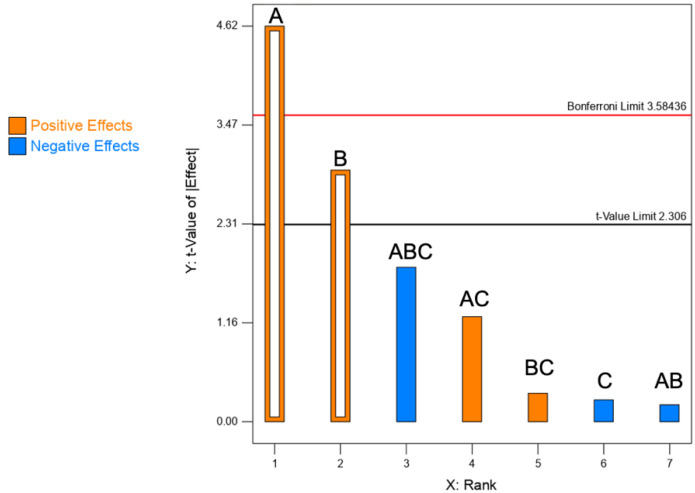
Pareto chart illustrating the rank of the t-values corresponding to the effect on spray drying yield of each factor and their interactions (empty bars: significant; full bars: non-significant). Blue bars = negative effects; orange bars = positive effects. A: CaPs concentration.; B: mannitol concentration; C: feed rate. The red line corresponds to the Bonferroni limit and the black one to the t-value limit.

As per Table 2, the yield of the process varied from 34.4 to 85.0%, which reveals an important dependence from the levels of the selected factors. ANOVA test shows a significant effect by both formulation factors, i.e., CaPs and mannitol concentrations (Table 3). In contrast, the process factor feed rate (factor C) was not significant, thus excluded from the analysis. More specifically, as illustrated in the Pareto chart of Figure 1, by increasing the concentration of CaPs (factor A) and mannitol (factor B), the yield was positively affected. Similarly, Dormenval et al. referred that, among the factors of importance for the manufacturing of spray-dried siRNA-loaded lipid nanoparticles for inhalation, higher feedstock concentrations increased the yield [8].

The yield was essentially affected by the sticking of particles to the drying chamber, cyclone walls and filter [9]. Finally, it must be underlined that the yield is not always an easy to interpret response. For instance, higher order models may govern the effect of the selected factors on the yield [10].

#### 3.1.2. Moisture Content

The measured moisture contents of the 11 powders produced are shown in Table 2.

The moisture content has been selected as CQA since it favours the agglomeration of very small particles, worsening powder flow properties, and affecting aerosolization. Moreover, it could also influence long-term physical and chemical stability [11]. In this study, the moisture content of all powders ranged between 1.2 and 4.0% (*w*/*w*). The lowest value of moisture content was achieved at a high level of mannitol concentration; mannitol is known to be a non-hygroscopic substance. Conversely, the highest moisture was obtained at a low level of mannitol.

**Figure 2:**
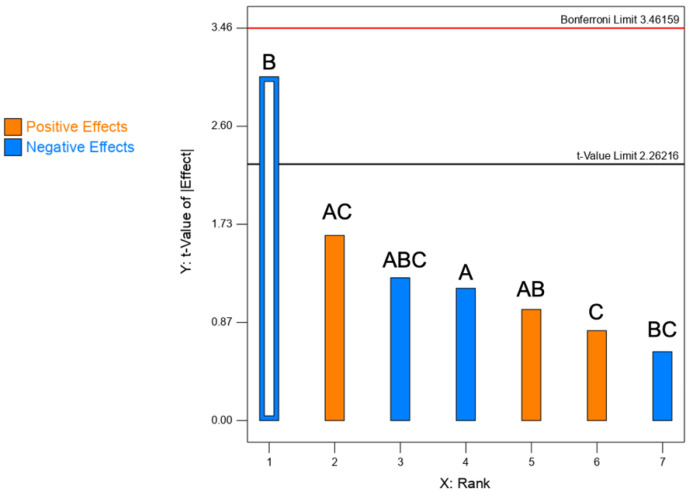
Pareto chart illustrating the rank of the t-values corresponding to the effect of each factor on moisture content. Empty bars: significant; full bars: non-significant. Blue bars = negative effects; orange bars = positive effects. A: CaPs conc.; B: mannitol conc.; C: feed rate. The red line corresponds to the Bonferroni limit and the black one to the t-value limit.

Indeed, the Pareto chart (Figure 2) illustrates the level of significance of mannitol concentration in decreasing the moisture content of the dry powder formulations. The ANOVA test shows that the factors CaPs concentration and feed rate factors did not significantly affect the moisture content. (Table 4).

#### 3.1.3. Particle Size Distribution of Microparticulate Dry Powders

Particle size distribution of inhalation dry powders is one of the three characteristics determining the particle aerodynamic behaviour together with size, density and shape. The prepared dry powders showed D_v50_ between 1.6 μm and 3.5 μm, a range considered suitable for particle inhalation [12]. The highest Dv_50_ value resulted when the solid concentration of feed solution and the feed rate were at their maximum levels (Run 11). Conversely, the lowest D_v50_ value was measured when the factors CaP concentration and feed rate were at low values, with the mannitol concentration at its high value (Run 3).

Other authors pointed out [13] that the geometric size (d_g_) of the drying particle in relation to droplet solvent evaporation, is described by Equation (2):

(2) $$d_{g}=\sqrt[{3}]{\frac{c_{f}}{\rho_{p}}}d_{D}$$

where c_f_ is the feed solution concentration, ρ_p_ the particle density and d_D_ the droplet size. Droplet size was expected to be affected by increasing the feed rate since more liquid is dispersed at the nozzle. Thus, the increase in feed solution concentration and in droplet size due to high feed rate, led to larger dried particles, depending on the particle density. In addition, the increase of feed rate leads to lower outlet temperature which results in lower drying efficiency, generating larger particles as well. However, examining the ANOVA test results (Table 5), the model was found not significant (*p* value: 0.859). Likely, the variation of the output in terms of particle size was minimally influenced by the selected factors and their ranges. We attempted also to examine other size distribution parameters, i.e., Dv_90_ or Span, but in neither case the model was significant. In summary, the values of Dv_50_ are quite small, meaning that the PSD of the microparticle is a robust parameter in the manufacturing ranges selected.

#### 3.1.4. Aerodynamic Performance of Spray Dried Powders

**Figure 3:**
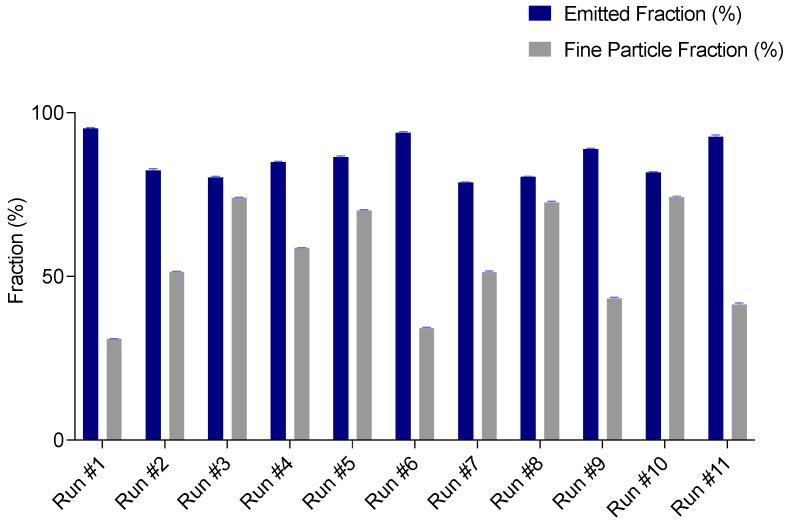
Emitted and Fine Particle Fraction of the eleven powders manufactured.

The Critical Quality Attributes selected to assess the in vitro powder aerodynamic performance of the powders were the Emitted Dose and the Fine Particle Dose. According to European Pharmacopeia, the Emitted Fraction should be not less than 75% of the dose metered in the device that, in this case, was 40.0 ± 0.5 mg. As reported in Figure 3, in all powders EF was higher than 75%. This means that all the formulations possessed favourable flow properties for aerosolization. However, the Emitted Dose response showed a non-significant effect of all the factors selected, thus ED was not further considered.

The fine particle aerodynamic size distribution is the indicator of the deposition behaviour of particles in the lung. Particles with a mean aerodynamic diameter between 1 to 5 μm are considered respirable [14]. FPD value measures the mass of aerosolized particles having an aerodynamic diameter lower than 5 μm. As it can be noticed in Table 2, FPD ranges from 11.8 to 24.3 mg, corresponding to Fine Particle Fraction of 31% and 74.3%, respectively (Figure 3).

The results of the ANOVA test (Table 6) shows that A (CaP concentration), C (feed rate) and their interaction AC are significant factors for respirability. The other terms (B, AB, BC, ABC) are insignificant and were not included in the model.

**Figure 4:**
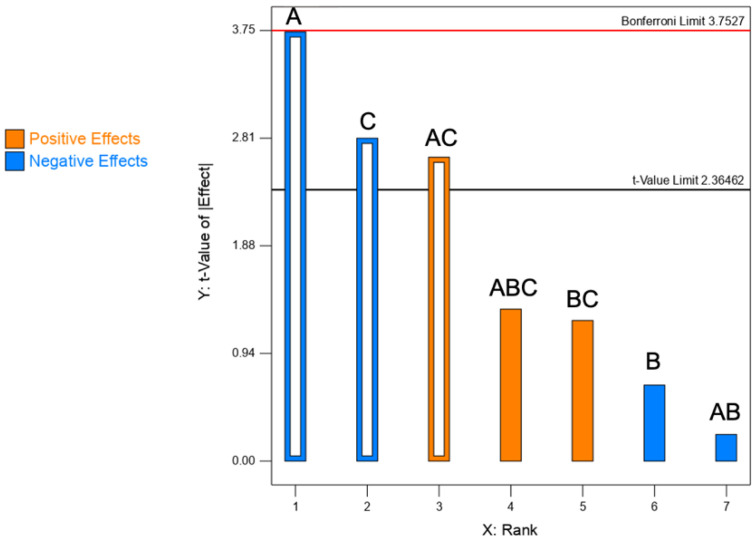
Pareto chart illustrating the rank corresponding to the effect of each factor on the FPD. Empty bars: significant; full bars: non-significant. Blue bars = negative effects; orange bars = positive effects. A: CaPs conc.; B: mannitol conc.; C: feed rate. The red line corresponds to the Bonferroni limit and the black one to the t-value limit.

Individually, factors A and C have a significant negative effect, that is a reduction of the fine particle dose, whereas their interaction has a positive effect (Pareto chart, Figure 4). Despite this was a curious situation, it is possible that two negative effects turn into a positive effect when they interact in the system. These results differ from those obtained from the PSD analysis, where the non-significant correlation of factors with geometric size was found. Besides geometric size, particle density and shape have a relevant impact on aerodynamic size and, subsequently, on FPD [15,16]. As an example, even though the powder of Run 5 has a D_v90_ of 7.7 μm, it exhibited the highest FPD value (24.3 mg); conversely, despite a more favourable D_v90_, the powder of Run 1 (D_v90_ 5.5 µm) showed the lowest FPD (11.8 mg).

#### 3.1.5. Z-Average of Nanoparticles Released in Water

Nanoparticle hydrodynamic diameter, d_H,_ recovered after microparticle dissolution is one of the critical quality attributes of this product. Thorley A. et al. [17] and Islam M.A et al. [18] have shown that the lower the d_H_, the easier the nanoparticle uptake by the cell. An in vitro test to assess the quality of the nanoparticles released from the dissolved microparticles was carried out. It is worth mentioning that this test did not aim to mimic the release of nanoparticles in vivo from inhaled microparticles deposited on the wet lung epithelium. Particle sizes of CaPs dispersion after microparticle dissolution in water are reported in Table 2. The results of the ANOVA test (Table 7) show a significant effect of the formulation factors CaPs and mannitol concentrations, together with their interaction. In contrast, the feed rate was insignificant, thus removed from the model.

**Figure 5:**
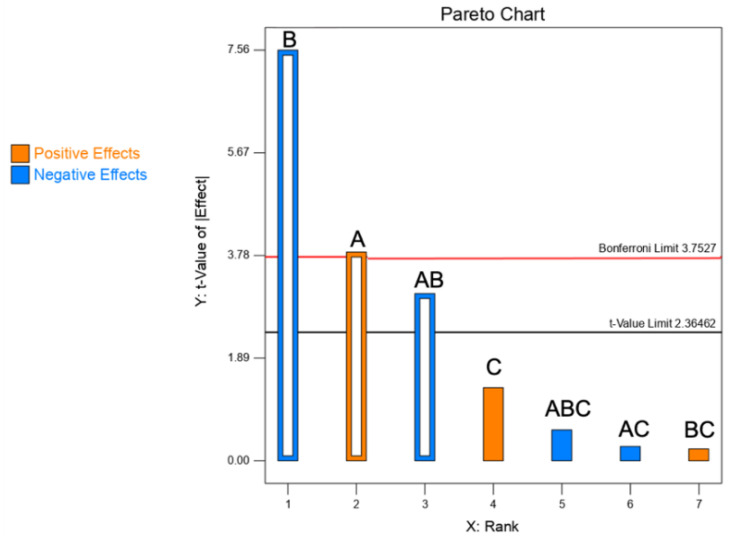
Pareto chart illustrating the rank corresponding to the effect of each factor on the Z-average diameter of restored nanoparticles. Empty bars: significant; full bars: non-significant. Blue bars = negative effects; orange bars = positive effects. A: CaPs conc.; B: mannitol conc.; C: feed rate. The red line corresponds to the Bonferroni limit and the black one to the t-value limit.

In the Pareto chart (Figure 5), the increase of CaPs concentration in the liquid feed (factor A) shows a positive effect, i.e., the size of the released nanoparticles rises. In contrast, the increase of mannitol concentration (factor B) exhibits a very significant negative effect, i.e., the size of released nanoparticles remains small. Since the CaPs size depends on nanoparticle agglomeration, the presence of mannitol reduces the likelihood of direct contact between adjacent nanoparticles. The polyol fills the space among the nanoparticles, enlarging the interparticle distance limiting the attractive forces. Thus, a high mannitol concentration helps to preserve the original size of CaPs nanoparticles. Keil et al. [19] formulated the hypothesis that a large amount of water lost from the nanoparticles’ dispersion during the spray drying process, may lead to the need for larger amounts of matrix/filler excipients.

In addition, a significant interaction between factors A and B was measured. In fact, the Pareto chart (Figure 5) showing the beneficial negative effect of AB interaction on the nanoparticle size upon release from the microparticles, supports the dependence of restored size on the CaPs and mannitol concentration ratio.

As the interaction between the two formulation factors (AB) was significant (Figure 5), we deduced that the relevant parameter for CaP nanoparticle size maintenance is the ratio between mannitol and nanoparticle amount. In fact, the highest size value of the released nanoparticles was measured when the ratio between nanoparticles and mannitol was 14:1, whereas the original small size of nanoparticles was maintained at the ratio 1:4.

Together with the nanoparticle size, for sake of completeness, we reported in the Supplementary material (Table S1) the polydispersity index and ζ-potential values. ζ-potential ranged from −13.0 to −21.2 mV, confirming the achievement of relatively stable colloidal systems.

### 3.2. Particle Morphology of DoE Powders

To understand the mechanisms of microparticle aerodynamics and nanoparticle release behaviour, i.e., the two most relevant characteristics for the heart targeting by inhalation of nanoparticles, it is imperative to look at their microscopical structure. The scanning electron microscopy coupled with a focused ion beam was used to visualize the particle size, shape, surface morphology and internal structures of microparticles. These micromeritic properties are key factors impacting on flow, dispersibility, and aerodynamic behaviour particle size of dry powders.

Beforehand, mannitol alone was spray-dried and the resulting particles examined to understand the impact of the polyol on particle structure. Then, powders from Runs 2, 3, and 5, were considered because they were very different from the others.

**Figure 6:**
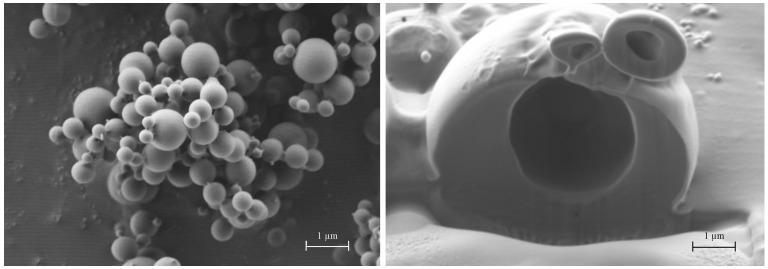
SEM plan-view micrograph of spray-dried mannitol microparticles (**left**) and cross-section of FIB-cut mannitol particle (**right**).

In Figure 6, SEM/FIB images illustrate plan-view and cross-section of FIB-cut microparticles of spray-dried mannitol alone. The dimensional range of the particles is between 200 nm and 1 μm, a quite narrow PSD. Their shape is rather spherical with a smooth surface. Similar morphology of mannitol microparticles has been described elsewhere [20]. In addition, FIB-cut allows observing the internal structure of these particles (Figure 6, right). The particle is hollow with a centred large cavity of around 1.0 µm, whereas the structure of the wall is dense and homogeneous.

When CaPs were added, their ratio to mannitol in the dispersion to spray dry changed the morphology of the resulting mannitol microparticles, now embedding CaPs. Torge et al. [21] investigated the morphology and aerodynamic properties of spray-dried mannitol microparticles embedding polylactic-co-glycolic acid nanoparticles at three different ratios. They found that mannitol content influenced morphology, nevertheless, all powders were suitable for pulmonary delivery.

**Figure 7:**
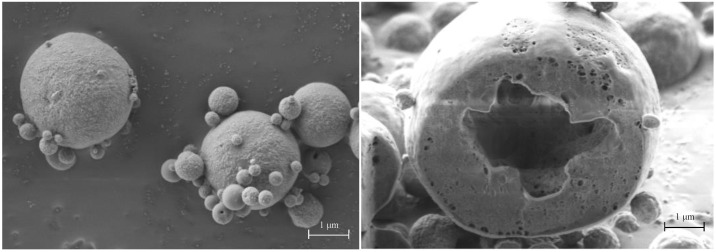
SEM plan-view micrograph of spherical microparticles embedding calcium phosphate nanoparticles from Run 3 (**left**) and cross-section of FIB-cut particle (**right**). Ratio CaPs: mannitol 1:4.

In Figure 7, particle shape, surface morphology and internal structure of microparticles from Run 3 (CaPs concentration 0.5 mg/mL; mannitol 2.0 mg/mL; feed rate: 3.5 mL/min) are displayed. The CaPs:mannitol ratio was 1:4. In this case, the microparticles had D_v10_*,* 1.3 µm and D_v90_ of 3.1 µm as volume diameter. The particle shape is spherical and close to that of spray-dried mannitol. However, the particle surface appears rougher, while the structure still exhibits an internal cavity. Differently, small pores are present in the wall around the cavity. An increase in the particle porosity leads to a decrease in particle density that reduces the aerodynamic particle size. The true density of this powder was 1.590 ± 0.002 g/cm^3^ and the apparent tapped density was 0.750 ± 0.002 g/cm^3^. Although the measured value of tapped density was higher than the 0.4 g/cm^3^ limit value according to Chougule et al. [22], respirable particles were prepared with this ratio of components.

**Figure 8:**
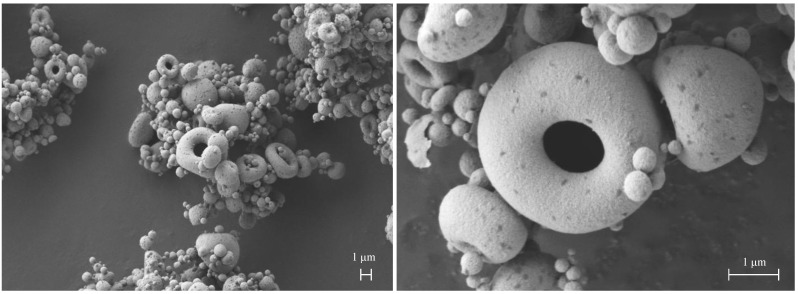
SEM plan-view micrographs of doughnut-shaped microparticles CaPs: mannitol 14:1 embedding unloaded calcium phosphate nanoparticles (Run 2).

In Run 2, a feed solution containing 0.5 mg/mL of mannitol dissolved in the dispersion containing 7.0 mg/mL of nanoparticles was dried at a feed rate of 3.5 mL/min. In Figure 8, SEM micrographs at different magnifications show microparticles with different sizes and shapes, mostly below 5 μm. Most of them are spheroidal, typical of particles prepared by spray drying [23]. However, doughnut-shaped structures of spray-dried microparticles (Figure 8) appeared in this composition in which nanoparticle concentration was high and mannitol low. Depending on their size, some microparticles have collapsed during formation. In fact, the larger particles show a central hole which makes them resemble a doughnut [24].

We speculated that the increase of nanoparticle fraction in the composition leads to a high Péclet number, i.e., the ratio of solvent evaporation rate (k) and diffusion coefficient of solute component *i* in liquid phase (D_i_), as shown in Equation (3) [25].


(3) $${Pe}_{i}=\frac{k}{{8D}_{i}}$$

**Figure 9:**
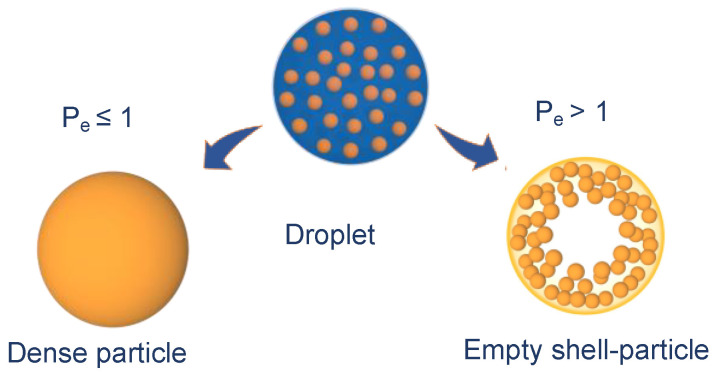
Drying of droplets containing particles of low (**left**) and high (**right**) Péclet number.

This dimensionless number is calculated from the ratio between the droplet surface area shrinking rate during drying and the rate of solute diffusion (nanoparticles and mannitol molecules) from the air/liquid interface to the droplet centre [26]. P_e_ drives the microparticle structure to be a dense, or rather hollow “empty particle” (Figure 9). Calcium phosphate nanoparticles have a diffusion coefficient in water lower than mannitol (2.7 × 10^−7^ cm^2^/s vs. 2.9 × 10^−6^ cm^2^/s) [27,28] Therefore, during droplet evaporation, a porous shell of nanoparticles is formed, allowing the residual core solvent to evaporate.

In this case, the morphology of the dried particles depends on the chemical nature, concentration, mechanical properties of the materials forming the shell, and the nanoparticle colloidal interactions [29,30]. The accumulation of nanoparticles at the particle surface during drying, in presence of a low amount of mannitol, weakened the resistance of the shell curvature. Depending on size, the particle in formation resists and remains spherical or collapses to form the hollow structures shown in Figure 8. However, the doughnut-like microparticle structures exhibited acceptable respirability, lower than spherical particles, still with a Fine Particle Fraction higher than 50%. Conversely, once dissolved in water, the doughnut-shaped microparticles released nanoparticles of increased size owing to the presence of clusters, deriving from the higher concentration of nanoparticles compared to mannitol (components’ ratio 14:1).

**Figure 10:**
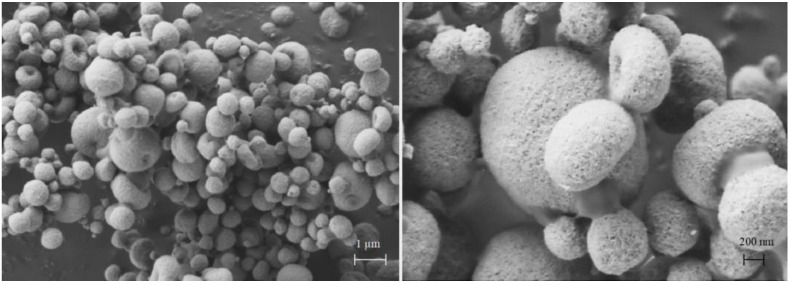
SEM plan-view micrographs of spray-dried microparticles from Run 5 embedding unloaded nanoparticles, ratio CaPs: mannitol 1:1.

In Figure 10, SEM pictures from Run 5 powder obtained with a CaPs:mannitol ratio of 1:1 are shown.

In this case, particles show a rough surface and dimpled shape due to an incomplete doughnut formation. The higher amount of mannitol in the composition makes the shell formed during drying more resistant, compared to microparticles of Figure 8. The results confirm the effect of the CaPs and mannitol ratio on the resistance of particle shells. The evident roughness of microparticles from Run 5 may explain the FPF that was about 70%. Surface roughness decreases the contact area between adjacent microparticles, thus reducing the inter-particulate cohesive forces [31]. This observation is in agreement with Chew et al. who demonstrated how the increase in surface roughness of spray-dried bovine serum albumin particles can result in enhancement of the aerodynamic performance, in terms of fine particle fraction [32]. However, the size of the nanoparticles released in water from Run 5 powder remained high.

## 4. Conclusions

In this work, we have studied the preparation by spray drying of inhalable mannitol microparticles embedding nanoparticles of calcium phosphate. The ratio of mannitol: nanoparticle content is the parameter that increases the respirability of the powder but, more significantly, protects the size of the released nanoparticles upon mannitol dissolution. The feed rate parameter, which is considered relevant for the yield of an industrial manufacturing process, was less significant for the characteristics of the microparticles obtained. However, in certain manufacturing conditions, the interaction of this process-related factor with the nanoparticle concentration was observed to be beneficial for microparticle respirability.

**Figure 11:**
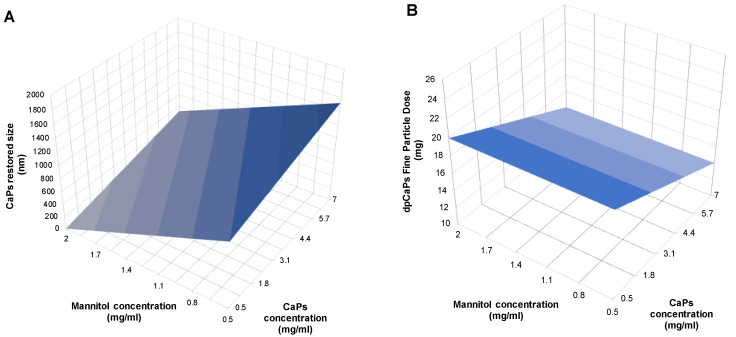
3D graphs of nanoparticle/mannitol concentration and restored nanoparticles size (**A**) or Fine Particle Dose of microparticles (**B**) at fixed spray drying feed rate of 5.25 mL/min.

The plots of Figure 11A,B quantify the effect of nanoparticle and carrier contents and their ratio on the released nanoparticles size and fine particle dose of microparticles. Increasing the mannitol concentration vs. the CaPs amount, increased the microparticle respirability and, more evidently, preserved the size of released nanoparticles. These quality attributes are crucial for the use of microparticles embedding nanoparticles for targeting the lung first and then the heart.

Taking up the observations relating to the morphology of microparticles embedding CaP nanoparticles, the concentration of the nanoparticles in the droplet subjected to drying led to microparticle structures that changed from spherical to dimpled and eventually to doughnut. This depends on the ratio between CaPs and mannitol as well. The respirability of these structures do not change too much, but the size of nanoparticles released upon dissolution of the carrier largely increases as the mannitol content is reduced. In this composite system for nanoparticle release in the lung, mannitol acts as a carrier capable of regulating the respirability but, more importantly, protecting the size of the released nanoparticles.

Finally, it is worth noticing that this work started from a pilot batch of nanoparticles done in an industrial environment. Spray drying at the industrial level has been already carried out with positive results.

## 5. Patents

Part of this research work is included in the Patent Application N°102020000021292-2020; Catalucci D, Iafisco M, Colombo P, Quarta E.

## Figures and Tables

**Table 1:** Matrix of the full-factorial design.

Std	Run	Factor A CaPs Concentration (mg/mL)	Factor B Mannitol Concentration (mg/mL)	Factor C Feed Rate (mL/min)
4	1	7.00	2.00	3.50
2	2	7.00	0.50	3.50
3	3	0.50	2.00	3.50
9	4	3.75	1.25	5.25
1	5	0.50	0.50	3.50
6	6	7.00	0.50	7.00
5	7	0.50	0.50	7.00
10	8	3.75	1.25	5.25
7	9	0.50	2.00	7.00
11	10	3.75	1.25	5.25
8	11	7.00	2.00	7.00

**Table 2:** Summary of the selected factors with the coded representation and responses of the eleven powders prepared.

	Factors			Responses					
Run	A CaPs (mg/mL)	B Mannitol (mg/mL)	C Feed Rate (ml/min)	Yield (%)	Moisture Content (%)	Z-Average Diameter (nm)	D_v50_ (μm)	ED (mg)	FPD (mg)
1	+1	+1	−1	84.4	1.2 ± 0.1	313.4 ± 7.0	3.0 ± 0.1	38.1 ± 0.2	11.8 ± 0.2
2	+1	−1	−1	62.5	1.3 ± 0.1	1787.0 ± 15.0	2.1 ± 0.1	33.0 ± 0.4	17.0 ± 0.3
3	−1	+1	−1	58.4	1.5 ± 0.1	85.4 ± 6.0	1.6 ± 0.1	32.1 ± 0.3	23.8 ± 0.4
4	0	0	0	85.0	3.2 ± 0.2	400.0 ± 7.0	2.6 ± 0.2	34.0 ± 0.2	20.0 ± 0.3
5	−1	−1	−1	53.2	4.0 ± 0.2	839.5 ± 6.0	3.1 ± 0.1	34.6 ± 0.3	24.3 ± 0.2
6	+1	−1	+1	75.2	3.6 ± 0.1	1992.0 ± 21.0	3.3 ± 0.2	37.6 ± 0.2	12.9 ± 0.2
7	−1	−1	+1	34.4	3.2 ± 0.1	957.4 ± 4.0	2.3 ± 0.2	31.5 ± 0.2	16.2 ± 0.3
8	0	0	0	72.2	2.8 ± 0.1	892.0 ± 8.0	3.0 ± 0.1	32.2 ± 0.1	23.4 ± 0.3
9	−1	+1	+1	61.8	1.4 ± 0.1	428.0 ± 7.0	2.8 ± 0.2	35.6 ± 0.1	15.4 ± 0.3
10	0	0	0	85.0	2.5 ± 0.2	423.0 ± 6.0	2.9 ± 0.1	32.7 ± 0.2	24.3 ± 0.2
11	+1	+1	+1	81.8	1.5 ± 0.2	418.4 ± 10.0	3.5 ± 0.1	37.1 ± 0.4	15.4 ± 0.4

**Table 3:** ANOVA results for the selected factorial model for the yield of the spray drying process.

Source	Sum of Squares	df	Mean Square	F-Value	*p*-Value	
Model	1621.05	2	810.53	15.02	0.0029	significant
A-CaPs conc.	1154.40	1	1154.40	21.39	0.0024	
B-Mannitol conc.	466.65	1	466.65	8.65	0.0217	
Curvature	613.66	1	613.66	11.37	0.0119	
Residual	377.85	7	53.98			
Lack of Fit	268.63	5	53.73	0.98	0.5738	not significant
Pure Error	109.23	2	54.61			
Cor Total	2612.57	10				

**Table 4:** ANOVA results for the moisture content of microparticles embedding nanoparticles.

Source	Sum of Squares	df	Mean Square	F-Value	*p*-Value	
Model	5.28	1	5.28	9.20	0.0162	significant
B-Mannitol conc.	5.28	1	5.28	9.20	0.0162	
Curvature	0.84	1	0.84	1.46	0.2608	
Residual	4.59	8	0.57			
Lack of Fit	4.35	6	0.72	5.88	0.1526	not significant
Pure Error	0.25	2	0.12			
Cor Total	10.72	10				

**Table 5:** ANOVA results for the selected factorial model for D_v50_.

Source	Sum of Squares	df	Mean Square	F-Value	*p*-Value	
Model	0.95	5	0.18	0.35	0.8590	not significant
A-CaPs conc	0.03	1	0.03	0.06	0.8214	
B-Mannitol conc	0.00	1	0.00	0.02	0.8920	
C-Feed rate	0.00	1	0.00	0.00	0.9639	
AC	0.45	1	0.45	0.84	0.4116	
BC	0.45	1	0.45	0.84	0.4116	
Curvature	0.00	1	0.00	0.01	0.9051	
Residual	2.15	4	0.54			
Lack of Fit	0.21	2	0.11	0.11	0.9013	not significant
Pure Error	1.94	2	0.97			
Cor Total	3.11	10				

**Table 6:** ANOVA results for the selected factorial model for FPD.

Source	Sum of Squares	df	Mean Square	F-Value	*p*-Value	
Model	131.97	3	43.99	9.64	0.0104	significant
A-CaPs conc	63.84	1	63.84	13.99	0.0096	
C-Feed rate	36.12	1	36.13	7.92	0.0306	
AC	32.00	1	32.00	7.01	0.0381	
Curvature	65.20	1	65.20	14.29	0.0092	
Residual	27.38	6	4.56			
Lack of Fit	17.09	4	4.27	0.83	0.6103	not significant
Pure Error	10.29	2	5.14			
Cor Total	224.55	10				

**Table 7:** ANOVA results for the selected factorial model for Z-average diameter.

Source	Sum of Squares	df	Mean Square	F-Value	*p*-Value	
Model	3.339 × 10^6^	3	1.113 × 10^6^	27.10	0.0007	significant
A-CaPs conc.	6.053 × 10^5^	1	6.053 × 10^5^	14.74	0.0086	
B-Mannitol conc.	2.344 × 10^6^	1	2.344 × 10^6^	57.10	0.0003	
AB	3.888 × 10^5^	1	3.888 × 10^5^	9.47	0.0217	
Curvature	1.722 × 10^5^	1	1.722 × 10^5^	4.19	0.0865	
Residual	2.644 × 10^5^	6	41060.16			
Lack of Fit	92176.29	4	23044.07	0.30	0.8600	not significant
Pure Error	1.542 × 10^5^	2	77092.33			
Cor Total	3.757 × 10^6^	10				

## Data Availability

Not applicable.

## Supplementary Materials

The following are available online at https://www.mdpi.com/article/10.3390/pharmaceutics13111825/s1; Table S1. Polydispersity index (PdI) and ζ-Potential of dispersed CaPs after powder dissolution in water.
