# Supplementary material for: Inhalable Microparticles Embedding Calcium Phosphate Nanoparticles for Heart Targeting: The Formulation Experimental Design
Source: Pharmaceutics. 2021 Nov 1;13(11):1825. doi: 10.3390/pharmaceutics13111825 (PMC8617656; doi:10.3390/pharmaceutics13111825)
Supplement: Supplementary file 1 [file pharmaceutics-13-01825-s001.zip › pharmaceutics-1414927-supplementary.pdf]

pharmaceutics

Supplementary Materials: Inhalable Microparticles Embedding Calcium Phosphate Nanoparticles for Heart Targeting: The Formulation Experimental Design

Eride Quarta, Fabio Sonvico, Ruggero Bettini, Claudio De Luca, Alessandro Dotti, Daniele Catalucci, Michele Iafisco, Lorenzo Degli Esposti, Gaia Colombo, Giovanna Trevisi, Dimitrios M. Rekkas, Alessandra Rossi, Tin Wui Wong, Francesca Buttini and Paolo Colombo

Table S1. Polydispersity index (PdI) and ζ-Potential of dispersed CaPs after powder dissolution in water.

Run	A	B	C	PdI	ζ–potential (mV)
1	+1	+1	−1	1.0	−21.0
2	+1	−1	−1	1.0	−21.2
3	−1	+1	−1	0.5	−18.3
4	0	0	0	1.0	−16.4
5	−1	−1	−1	0.4	−16.0
6	+1	−1	+1	0.3	−16.5
7	−1	−1	+1	0.6	−16.4
8	0	0	0	0.9	−15.7
9	−1	+1	+1	0.6	−16.5
10	0	0	0	0.8	−16.0
11	+1	+1	+1	0.2	−13.0
